# Effects of 6-month eicosapentaenoic acid treatment on postprandial hyperglycemia, hyperlipidemia, insulin secretion ability, and concomitant endothelial dysfunction among newly-diagnosed impaired glucose metabolism patients with coronary artery disease. An open label, single blinded, prospective randomized controlled trial

**DOI:** 10.1186/s12933-016-0437-y

**Published:** 2016-08-26

**Authors:** Takahiro Sawada, Hideo Tsubata, Naoko Hashimoto, Michinori Takabe, Taishi Miyata, Kosuke Aoki, Soichiro Yamashita, Shogo Oishi, Tsuyoshi Osue, Kiminobu Yokoi, Yasue Tsukishiro, Tetsuari Onishi, Akira Shimane, Yasuyo Taniguchi, Yoshinori Yasaka, Takeshi Ohara, Hiroya Kawai, Mitsuhiro Yokoyama

**Affiliations:** 1 Division of Cardiovascular Medicine, Department of Internal Medicine, Hyogo Prefectural Himeji Cardiovascular Center, 520 Saisho-Kou, Himeji, Hyogo 670-0981 Japan; 2 Division of Diabetes and Endocrinology, Hyogo Prefectural Himeji Cardiovascular Center, 520, Saisho-Kou, Himeji, Hyogo 670-0981 Japan

**Keywords:** Eicosapentaenoic acid, Impaired glucose metabolism, Postprandial hyperglycemia, Postprandial insulin secretion, Postprandial hypertriglyceridemia, Endothelial dysfunction

## Abstract

**Background:**

Recent experimental studies have revealed that n-3 fatty acids, such as eicosapentaenoic acid (EPA) regulate postprandial insulin secretion, and correct postprandial glucose and lipid abnormalities. However, the effects of 6-month EPA treatment on postprandial hyperglycemia and hyperlipidemia, insulin secretion, and concomitant endothelial dysfunction remain unknown in patients with impaired glucose metabolism (IGM) and coronary artery disease (CAD).

**Methods and results:**

We randomized 107 newly diagnosed IGM patients with CAD to receive either 1800 mg/day of EPA (EPA group, n = 53) or no EPA (n = 54). Cookie meal testing (carbohydrates: 75 g, fat: 28.5 g) and endothelial function testing using fasting-state flow-mediated dilatation (FMD) were performed before and after 6 months of treatment. The primary outcome of this study was changes in postprandial glycemic and triglyceridemic control and secondary outcomes were improvement of insulin secretion and endothelial dysfunction. After 6 months, the EPA group exhibited significant improvements in EPA/arachidonic acid, fasting triglyceride (TG), and high-density lipoprotein cholesterol (HDL-C). The EPA group also exhibited significant decreases in the incremental TG peak, area under the curve (AUC) for postprandial TG, incremental glucose peak, AUC for postprandial glucose, and improvements in glycometabolism categorization. No significant changes were observed for hemoglobin A1c and fasting plasma glucose levels. The EPA group exhibited a significant increase in AUC-immune reactive insulin/AUC-plasma glucose ratio (which indicates postprandial insulin secretory ability) and significant improvements in FMD. Multiple regression analysis revealed that decreases in the TG/HDL-C ratio and incremental TG peak were independent predictors of FMD improvement in the EPA group.

**Conclusions:**

EPA corrected postprandial hypertriglyceridemia, hyperglycemia and insulin secretion ability. This amelioration of several metabolic abnormalities was accompanied by recovery of concomitant endothelial dysfunction in newly diagnosed IGM patients with CAD.

*Clinical Trial Registration* UMIN Registry number: UMIN000011265 (https://www.upload.umin.ac.jp/cgi-open-bin/ctr/ctr.cgi?function=brows&action=brows&type=summary&recptno=R000013200&language=E)

**Electronic supplementary material:**

The online version of this article (doi:10.1186/s12933-016-0437-y) contains supplementary material, which is available to authorized users.

## Background

Type 2 diabetes mellitus (DM) is a prevalent disease and recognized as a major risk factor for coronary artery disease (CAD) [1, 2]. Epidemiological studies and meta-analyses indicate that dietary modifications that increase the relative abundance of dietary n-3 polyunsaturated fatty acids (PUFA), such as eicosapentaenoic acid (EPA) and docosahexaenoic acid, can reduce the risks of DM as well as CAD [3–5] Most animal experiments also document beneficial effects of n-3 PUFA on insulin sensitivity, secretion, and glucose metabolism under condition of obesity, insulin resistance and DM [6, 7]. These findings suggest that n-3 PUFA may help control and prevent DM possibly due to improvement of insulin secretion and sensitivity.

However, data from clinical trials have been conflicting. Some studies indicated that n-3 PUFA improved insulin sensitivity in humans, whereas others found that n-3 PUFA had no insulin sensitizing effects [8]. Laria et al. reported that dietary EPA and docosahexaenoic acid did not alter peripheral insulin sensitivity, postprandial glucose disposal, or insulin secretion in insulin-resistant, non-diabetic overweight individual [9]. In contrast, a randomized clinical trial in overweight patients with DM revealed a beneficial effect of purified EPA on glucose homeostasis and insulin sensitivity [10]. Thus, the effect of EPA on the prevention or exacerbation of DM still remains unknown and has not yet been studied in clinical intervention trials in high-risk patients with impaired glucose metabolism (IGM). We hypothesized that long-term EPA treatment would predominantly improve postprandial state of insulin secretion and postprandial hyperglycemia and hypertriglyceridemia, and lead to prevent development of DM in patients with newly diagnosed IGM. In addition, these metabolic effects would be associated with improvement in concomitant endothelial dysfunction. Therefore, the present study was designed to examine the effects of EPA on postprandial plasma metabolic parameters and fasting endothelial function among newly diagnosed IGM patients with CAD.

## Methods

### Participants

This study was open label, single blinded, randomized controlled study approved by the Ethics Committee of Hyogo Prefectural Himeji Cardiovascular Center and complied with the Declaration of Helsinki. Informed written consent was obtained from all eligible patients before randomization to either EPA treatment or non-EPA treatment groups. This study is registered in the UMIN Clinical Trials Registry under the identifier UMIN000011265 (https://www.upload.umin.ac.jp/cgi-open-bin/ctr/ctr.cgi?function=brows&action=brows&type=summary&recptno=R000013200&language=E).

All potential participants who had documented chronic CAD, and with hemoglobin A1c levels <6.5 % (using the National Glycohemoglobin Standardization Program method), fasting plasma glucose (PG) levels ≥116 mg/dL or fasting plasma triglyceride (TG) levels ≥150 mg/dL by screening laboratory examination, were recruited from outpatients between July 2013 and December 2014. CAD was defined as stenosis of >50 % of the diameter of a coronary artery on angiography or computed tomography coronary angiography, or a history of myocardial infarction, percutaneous coronary intervention, or coronary artery bypass surgery. They underwent a 75-g oral glucose tolerance test (OGTT) and were diagnosed with DM or impaired glucose tolerance (IGT) according to Japan Diabetic Society criteria (DM: fasting PG ≥126 mg/dL and PG ≥200 mg/dL at 2 h after the OGTT; IGT: PG ≥140 mg/dL at 2 h after the OGTT). Patients were eligible to participate if they had newly diagnosed IGM (defined as IGT or DM during the OGTT). The exclusion criteria were presence of established DM, treatment history by anti-diabetic agent, systemic disease, including hepatic disease, renal disease (serum creatinine levels ≥2.5 mg/dL), collagen disease, infection, or malignancy and acute coronary syndrome.

**Fig. 1:**
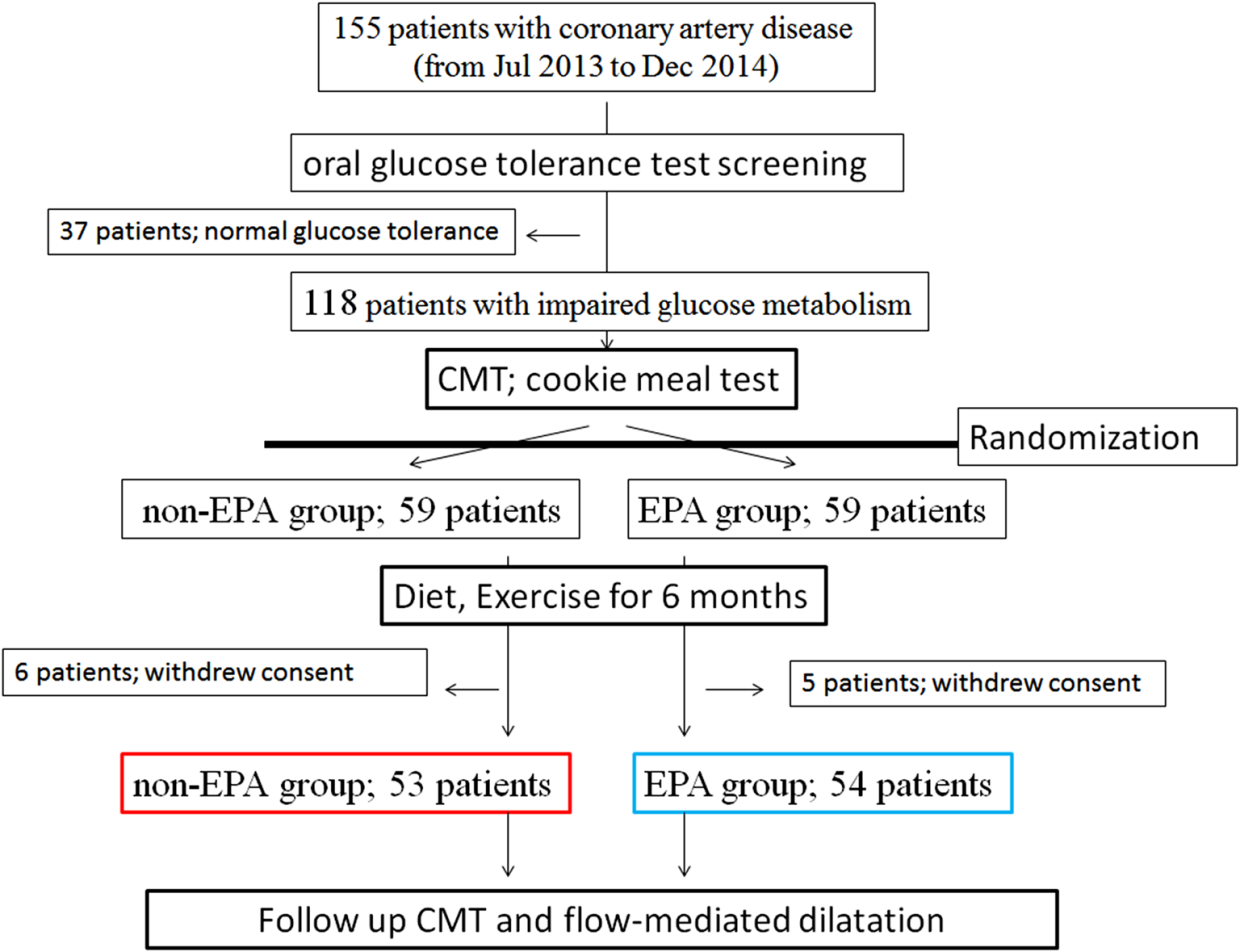
Flow chart of patient recruitment and participation

As shown in Fig. 1, 155 patients underwent OGTT screening, and 118 patients were diagnosed with IGM. At enrollment, all patients met at least once with a dietician for nutritional guidance and were encouraged to start and maintain a low-calorie diet and mild-to-moderate exercise levels. And then, 118 patients were randomly assigned to the EPA group (59 patients) or the non-EPA group (59 patients). Randomization was performed by means of random, permuted blocks of four in sealed envelope. We used EPADEL soft capsule (Mochida Pharmaceutical Co. Ltd., Tokyo, Japan) containing 900 mg of highly (≥98 %) purified EPA ethyl ester per capsule. In this drug product, EPA is actually purified from long-chain polyunsaturated fatty acids present in fish oil. We adopted the most widely used therapeutic dose of 900 mg of EPA ethyl ester capsule administered orally twice a day immediate after meals, as these dose of EPA has been shown to possess the beneficial effects in humans [11, 12].

All patients were followed without hypoglycemic drugs and insulin treatment during the study period. And all participants were also requested to maintain any concomitant drug treatment throughout the study. At each visit, we questioned the participants regarding any adverse events.

Anthropometric measurements (body weight and body mass index) and systolic and diastolic blood pressures were measured at randomization and after 6 months of treatment. Blood pressure was measured with the patient in the supine position after 15 min of rest.

### Blood biochemistry

Blood samples were collected from all patients after an overnight fast and were used to determine PG, hemoglobin A1c, 1,5-anhydro-glucitol, immune reactive insulin (IRI), total cholesterol, high-density lipoprotein cholesterol (HDL-C), low-density lipoprotein (LDL) cholesterol, TG, remnant-like particle cholesterol, and C-reactive protein. All biochemical analyses were performed using a commercially available kit. Hemoglobin A1c levels were measured using high-performance liquid chromatography. IRI concentrations were measured using a chemiluminescent enzyme immunoassay and remnant-like particle cholesterol was measured using an assay kit (Japan Immunoresearch Laboratories Co., Ltd., Takasaki, Japan). Plasma total fatty acid concentrations were measured by a central laboratory (BML Inc. Saitama, Japan), which was previously described [12, 13]. Plasma fatty acid composition was determined by capillary gas chromatography. Briefly, plasma lipids were extracted by Folch’s procedure, and then fatty acids were methylated with boron trifluoride and methanol, and methylated fatty acids were analyzed using the SHIMAZU GC-17A gas chromatograph (Shimazu Corporation, Kyoto, Japan) and a BPX70 capillary column (0.25 mm ID*30 m; SGE international Ltd., Melbourne, Australia) [13].

### Cookie meal test

A cookie meal test (CMT) was performed for glucose and fat loading at randomization and 6-month follow-up. The cookie consisted of carbohydrates (75 g; 85 % flour starch, 15 % maltose), butter fat (28.5 g), and protein (8 g) with 592 kcal (SARAYA Corp., Osaka, Japan). The CMT is sufficient for providing information regarding glucose intolerance and postprandial hypertriglyceridemia. The same values of cut-off point is proposed to be used at 2h PG for the evaluation of DM and IGT as WHO criteria for OGTT with the exception of exocrine pancreatic dysfunction [14]. Therefore, we used the CMT to simultaneously evaluate postprandial hyperglycemia (using the same criteria as for the OGTT) and postprandial hypertriglyceridemia.

All participants completed a 12-h overnight fast before the CMT. The cookie was ingested with water within a 20-min period, and blood samples for PG, IRI, and TG measurement were obtained at 0, 1 and 2 h after the participant had ingested half volume of the cookie [14]. These values were reported as PG-1h, PG-2h, IRI-1h, IRI-2h, TG-1h and TG-2h, respectively.

As an index of postprandial hyperglycemia, we calculated the incremental glucose peak [(the maximal incremental increase in PG at any point after meal loading)—(fasting PG)]. We also calculated the area under the response curves for PG (AUC-PG) and IRI (AUC-IRI) using the trapezoid rule. And then, we calculated AUC-IRI/AUC-PG as an index of postprandial insulin secretion ability. The homeostasis model assessment ratio (HOMA-R = fasting IRI × fasting PG/405) was used as an index of insulin resistance.

Incremental TG peak [(the maximal incremental increase in TG at any point after loading)—(fasting TG)] was calculated as an index of postprandial hypertriglyceridemia. The trapezoid rule was used to calculate the area under the TG-response curve (AUC-TG).

Small dense LDL particles, which are more susceptible to oxidation, have been suggested to be more atherogenic than large buoyant LDL [15, 16]. In this study, the TG/HDL-C ratio was used to assess the presence of small dense LDL particles [17].

### Measurement of endothelial function

We selected percent change in flow-mediated dilatation (FMD) as the surrogate marker for endothelial function [18–20] Before undergoing FMD testing, the participants were instructed to fast for >12 h and to abstain from any medications, smoking, alcohol, caffeine, and antioxidant vitamins during that time. All participants rested for at least 15 min in a seated position in a quiet dark air-conditioned room (22–25 °C) before the FMD measurements, which was previously described [18, 19]. In brief, a longitudinal image of the right or left brachial artery was recorded at baseline using high-resolution ultrasonography and a 10-MHz linear array transducer probe (UNEX, Nagoya, Japan). A forearm-cuff was inflated for 5 min at 50 mmHg above the systolic blood pressure just prior to FMD measurement. After cuff deflation, the diastolic diameter of the brachial artery was semi-automatically and continuously recorded for 2 min using software-equipped instrument that could monitor arterial diameter. The %FMD was estimated as the percent change in the vessel diameter, which corresponded to the maximum dilatation that was reached during reactive hyperemia divided by the baseline value. Because the %FMD value is highly dependent on the baseline diameter of the vessel, we compared the baseline diameters and absolute changes in the brachial artery’s diameters for each group. We have also previously confirmed that there is excellent intra- and inter-observed agreement for the %FMD measurement [18, 19].

### Primary and secondary endpoints

The primary endpoint of this study was postprandial changes in glucose concentration (incremental glucose peak) and postprandial changes in TG (incremental TG peak), derived from CMT after 6 months treatment of EPA. The secondary endpoints was improvement in postprandial insulin secretion ability and concomitant endothelial dysfunction.

### Statistical analysis

All statistical analyses were performed using MedCalc software (Version 9.3; Mariakerke, Belgium), and a two-tailed P < 0.05 was considered statistically significant.

Sample size was determined by power analysis using preliminary data obtained in our laboratory with the following assumptions: Type I error of 0.05 (two-tailed), power of 90 %, difference in the incremental glucose peak and incremental TG peak between before and after EPA treatment of 20 and 20 mg/dL, respectively, and a standard deviation of 30 and 25, respectively. Therefore, a minimum of 31 patients would yield 90 % power to detect a significant difference.

All variables were checked for normal distribution using Kolmogorov–Smirnov algorithm. Continuous variables with normal distribution were reported as mean ± standard deviation, otherwise as median and interquartile ranges. Inter-group comparisons of normal distributed data were performed using the unpaired Student’s t test, and Mann–Whitney test was used if the variables did not indicate normal distribution. Comparison of categorical variables was performed using χ^2^ test. Differences between the biochemical data with normal distribution before and after treatment were compared using a paired t test, otherwise using the Wilcoxon rank-sum test. Multivariable regression analyses were used to identify factors that were independently associated with the changes in incremental glucose peak and %FMD. Biologically plausible factors were included in the original model. Potential factors of interest with a univariate P ≤ 0.1 were entered into multivariate models.

## Results

### Study population

Among the 118 eligible patients, five patients in the EPA group and six patients in the non-EPA group withdrew their consent before second CMT. Therefore, the final analyses included data from 54 patients in the EPA group and 53 patients in the non-EPA group (Fig. 1).

During the study period, one patient in the EPA group and one patient in the non-EPA group underwent percutaneous coronary intervention due to progression of CAD. However, there were no major adverse events such as massive bleeding complication and gastrointestinal disorder requisite to treat that were related to the EPA treatment.

### Baseline characteristics, laboratory data, and anthropometric measurements

As shown in Table 1, the patients’ baseline characteristics and concomitant drug use were similar for groups. Most baseline laboratory findings were similar between the two groups, although fasting PG levels in the EPA group were lower than those in the non-EPA group. At baseline, resting brachial artery diameters and levels of endothelial function impairment were similar between the two groups. After 6 months treatment, both groups exhibited significant reductions in body weight and body mass index although there were no significant inter-group differences in these changes (Table 2).

**Table 1:** Comparison of baseline characteristics and concomitant use drugs between the two groups

	Non-EPA group (n = 53)	EPA group (n = 54)	P value
Age (y.o)	68.9 ± 8.8	67.8 ± 9.1	0.49
Sex (male, n, %)	43 (81.1 %)	44 (81.5 %)	0.84
Coronary risk factor			
Hypertension (n, %)	49 (92.5 %)	48 (88.9 %)	0.76
Dyslipidemia (n, %)	53 (100 %)	54 (100 %)	0.99
Smoking (n, %)	4 (7.5 %)	5 (9.3 %)	0.98
Concomitant use drug			
Statin (n, %)	44 (83.0 %)	46 (85.2 %)	0.97
Calcium channel blocker (n, %)	30 (56.6 %)	27 (50.0 %)	0.62
ACEI/ARB (n, %)	35 (66.0 %)	40 (74.1 %)	0.49

Values are presented as number (%) or mean ± standard deviation, as indicated

*ACEI* angiotensin converting enzyme inhibitor, *ARB* angiotensin receptor blocker

**Table 2:** Comparison of anthropometric analysis between baseline and 6 months, and comparison of absolute change from baseline between the two groups

Variable	Non-EPA group (n = 53)		EPA group (n = 54)		P value
	Baseline	6-month	Baseline	6-month	
Weight (kg)	67.8 ± 10.0	66.3 ± 9.9*	67.6 ± 11.2	66.4 ± 11.0*	0.97
Absolute Δ	−1.1 (−3.0, 0.0)		−1.0 (−2.8, 0.0)		0.49
Body mass index (kg/m^2^)	25.4 ± 2.4	24.8 ± 2.4*	25.3 ± 2.9	24.8 ± 2.8*	0.90
Absolute Δ	−0.5 (−1.1, 0.0)		−0.4 (−1.0, 0.0)		0.53
Systolic blood pressure (mmHg)	135.6 ± 15.0	133.0 ± 17.1	132.9 ± 19.5	129.7 ± 19.0	0.35
Absolute Δ	−3.0 (−13.0, 8.0)		−3.0 (−10.0, 4.0)		0.89
Diastolic blood pressure (mmHg)	78.6 ± 9.8	77.5 ± 10.9	77.2 ± 11.7	76.3 ± 8.2	0.51
Absolute Δ	−2.0 (−7.0, 3.3)		0.0 (−7.0, 6.0)		0.63

P values represent comparison of each values between groups at 6 months except for absolute Δ

As for absolute Δ of each values, P values represent comparison between the two groups

Values are presented as mean ± standard deviation or medians and interquartile ranges, as indicated

* P < 0.001 vs baseline

### Changes in fasting lipid and glucose profiles

The fasting lipid and glucose profiles at baseline and 6 months are shown in Table 3. After 6 months of treatment, the median EPA/arachidonic acid (AA) ratio significantly increased in the EPA group (from 0.31 to 1.08, P < 0.0001), and significantly increased HDL-C levels also occurred in the EPA group. Significant reductions in the levels of LDL cholesterol, TG, TG/HDL-C ratio, and remnant-like particle cholesterol were observed in the EPA group, but not in the non-EPA group. The EPA group exhibited significantly increased 1,5-anhydro-glucitol levels, although no changes were observed in the non-EPA group. The changes of TG, TG/HDL-C ratio and 1,5-anhydro-glucitol levels from baseline were also significantly larger in the EPA group than the non-EPA group. Neither group exhibited significant changes in fasting levels of PG, IRI, hemoglobin A1c, and HOMA-R.

**Table 3:** Comparison of biochemical data and flow-mediated dilatation data between baseline and 6 months, and comparison of absolute change from baseline between the two groups

Variable	Non-EPA group (n = 53)		EPA group (n = 54)		P value
	Baseline	6-month	Baseline	6-month	
Fasting PG (mg/dL)	111.2 ± 10.4	109.9 ± 10.8	105.8 ± 11.1^†^	104.5 ± 9.1	0.001
Absolute Δ	−1.0 (−6.0, 4.0)		−1.0 (−5.0, 4.0)		0.69
Hemoglobin A1c (NGSP; %)	6.1 ± 0.4	6.1 ± 0.4	6.0 ± 0.3	5.9 ± 0.4	0.02
Absolute Δ	0.0 (−0.1, 0.13)		0.0 (−0.1, 0.10)		0.49
1,5-anhydro-glucitol (μg/mL)	16.5 ± 6.1	16.1 ± 6.4	18.5 ± 7.8	19.3 ± 8.0*	0.02
Absolute Δ	−0.6 (−1.8, 1.2)		0.9 (−0.8, 1.9)		0.03
Fasting IRI (µU/mL)	6.4 ± 3.3	6.1 ± 2.9	6.6 ± 3.4	6.5 ± 3.0	0.49
Absolute Δ	−0.1 (−1.4, 0.7)		−0.2 (−2.0, 1.8)		0.63
HOMA-R	1.6 (1.0, 2.4)	1.6 (1.1, 2.0)	1.6 (1.0, 2.3)	1.6 (1.0, 2.2)	0.86
Absolute Δ	0.0 (−0.4, 0.2)		0.0 (−0.5, 0.5)		0.67
Total cholesterol (mg/dL)	171.7 ± 31.9	169.1 ± 27.7	171.8 ± 30.8	167.4 ± 29.4	0.76
Absolute Δ	−1.0 (−12.5, 8.5)		−3.5 (−13.0, 7.0)		0.45
LDL cholesterol (mg/dL)	96.7 ± 25.1	95.5 ± 23.3	97.9 ± 27.6	92.5 ± 26.8**	0.53
Absolute Δ	1.0 (−10.2, 8.0)		−4.0 (−13.0, 3.0)		0.09
HDL-C (mg/dL)	48.2 ± 9.8	48.2 ± 11.6	46.7 ± 9.3	50.1 ± 13.2**	0.51
Absolute Δ	0.0 (−4.3, 3.0)		2.0 (−3.0, 8.0)		0.05
TG (mg/dL)	123.3 ± 59.2	120.8 ± 62.2	137.8 ± 67.6	103.0 ± 38.1***	0.10
Absolute Δ	−3.0 (−35.0, 19.0)		−24.0 (−54.0, −3.0)		0.005
TG/HDL-C ratio	2.46 (1.43, 3.54)	2.18 (1.38, 3.54)	2.58 (1.65, 4.63)	2.02 (1.35, 2.65)***	0.39
Absolute Δ	−0.04 (−0.72, 0.33)		−0.73 (−1.44, −0.12)		0.0004
Remnant-like particle-cholesterol (mg/dL)	9.1 ± 6.1	8.4 ± 6.1	10.4 ± 6.4	7.3 ± 3.9***	0.26
Absolute Δ	−1.0 (−3.1, 1.1)		−1.7 (−4.8, −0.3)		0.07
EPA (μg/mL)	84.0 ± 50.7	81.1 ± 45.0	69.3 ± 40.7	196.4 ± 51.4***	<0.0001
Absolute Δ	−1.0 (−23.5, 24.0)		128.0 (98.0, 164.0)		<0.0001
AA (μg/mL)	205.6 ± 55.3	201.2 ± 48.1	206.3 ± 52.6	177.0 ± 39.7***	0.006
Absolute Δ	−2.0 (−34.8, 22.5)		−30.0 (−52.0, −3.0)		0.001
Docosahexaenoic acid (μg/mL)	162.5 ± 64.6	152.2 ± 57.7	158.9 ± 70.0	134.2 ± 48.1**	0.08
Absolute Δ	−18.0 (−44.3, 15.8)		−21.5 (−55.0, 12.0)		0.27
EPA/AA ratio	0.35 (0.25, 0.52)	0.36 (0.27, 0.46)	0.31 (0.21, 0.49)	1.08 (0.88, 1.45)***	<0.0001
Absolute Δ	−0.01 (−0.11, 0.10)		0.78 (0.52, 1.05)		<0.0001
Docosahexaenoic acid/AA	0.75 (0.59, 1.02)	0.74 (0.63, 0.96)	0.78 (0.53, 0.97)	0.77 (0.56, 0.95)	0.92
Absolute Δ	−0.01 (−0.19, 0.11)		−0.01 (−0.15, 0.11)		0.71
C-reactive protein (mg/dL)	0.09 (0.04, 0.14)	0.06 (0.04, 0.12)	0.10 (0.04, 0.22)	0.06 (0.03, 0.11)***	0.55
Absolute Δ	−0.01 (−0.05, 0.02)		−0.01 (−0.08, 0.00)		0.05
%FMD (%)	4.1 ± 1.8	4.0 ± 1.6	3.6 ± 1.7	5.2 ± 1.9***	0.0005
Absolute Δ	0.1 (−0.6, 0.8)		1.6 (0.7, 2.5)		<0.0001
Rest brachial artery diameter (mm)	4.27 ± 0.55	4.23 ± 0.58	4.23 ± 0.60	4.23 ± 0.58	0.72
Absolute change in brachial artery diameter (mm)	0.17 ± 0.07	0.17 ± 0.06	0.15 ± 0.07	0.22 ± 0.07***	0.0005
Absolute Δ	0.01 (−0.03, 0.03)		0.06 (0.02, 0.08)		<0.0001

P values represent comparison of each values between groups at 6 months except for absolute Δ

As for absolute Δ of each values, P values represent comparison between the two groups

Values are presented as mean ± standard deviation or medians and interquartile ranges, as indicated

*PG* plasma glucose, *IRI* immune reactive insulin, *HOMA*-*R* homeostasis model assessment ratio, *LDL* low-density lipoprotein, *HDL*-*C* high-density lipoprotein cholesterol, *TG* triglyceride, *EPA* eicosapentaenoic acid, *AA* arachidonic acid, *FMD* flow-mediated dilatation

^†^ P < 0.05 vs baseline values of non-EPA group

* P < 0.05 vs baseline, ** P < 0.01 vs baseline, *** P < 0.0001 vs baseline

### Changes in the CMT results

**Fig. 2:**
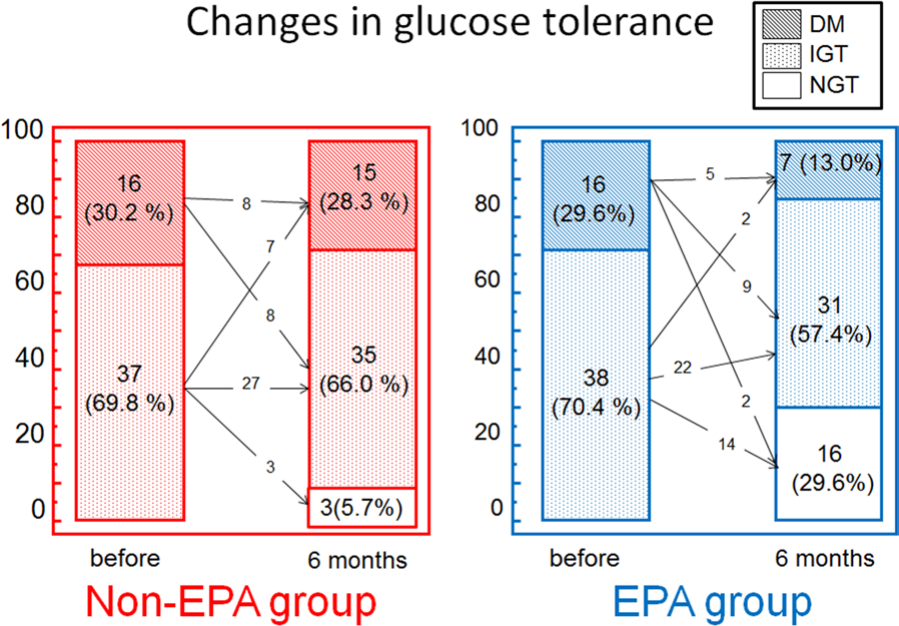
Categorical allocation according to glucose tolerance at baseline and after 6 months. *Numbers on the lines* represent the number of patients transferred between glucose categories. Deteriorated patients were significantly larger in the non-EPA group, while improved patients were significantly larger in the EPA group (deteriorated glucose tolerance; seven patents in the non-EPA group vs two patients in the EPA group, improved glucose tolerance; 11 patients in the non-EPA group vs 25 patients in the EPA group, p = 0.01). Comparison was performed using χ^2^ test

**Fig. 3:**
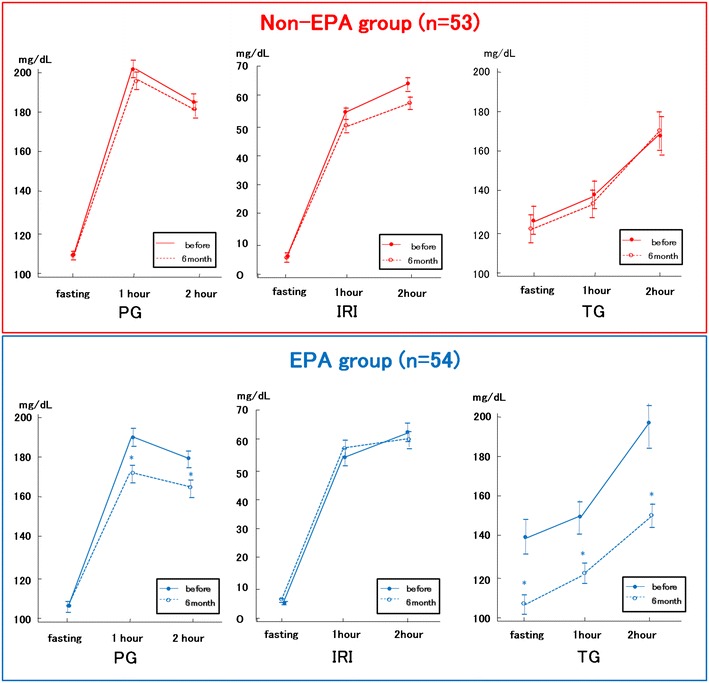
Plasma glucose (PG), immune reactive insulin (IRI), and triglyceride (TG) levels during cookie meal test at baseline and 6 months. *Bars* indicate SEM. * indicates P < 0.05 vs baseline. Comparisons of data between before and after treatment were performed using a paired t test

The CMT data from baseline and after 6 months of treatment are shown in Figs. 2, 3 and Table 4. The baseline CMT revealed that, among patients from the non-EPA group, 37 patients (69.8 %) had IGT and 16 patients (30.2 %) had DM. Similarly, among patients from the EPA group, 38 patients (70.4 %) had IGT and 16 patients (29.6 %) had DM (Fig. 2). After 6 months, the EPA group included 16 patients (29.6 %) with normal glucose tolerance, 31 patients (57.4 %) with IGT and seven patients (13.0 %) with DM. The non-EPA group included three patients (5.7 %) with normal glucose tolerance, 35 patients (66.0 %) with IGT and 15 patients (28.3 %) with DM. With regard to changes in glucometabolic category, deteriorated patients were significantly larger in the non-EPA group, while improved patients were significantly larger in the EPA group (deteriorated glucose tolerance; seven patents in the non-EPA group vs two patients in the EPA group, improved glucose tolerance; 11 patients in the non-EPA group vs 25 patients in the EPA group, p = 0.01).

**Table 4:** Comparison of cookie meal test data between baseline and 6 months, and comparison of absolute change from baseline between the two groups

Variable	Non-EPA group (n = 53)		EPA group (n = 54)		P value
	Baseline	6-month	Baseline	6-month	
Glucose tolerance test					
NGT (n, %)	0 (0.0 %)	3 (5.7 %)	0 (0.0 %)	16 (29.6 %)**	0.001
IGT (n, %)	37 (69.8 %)	35 (66.0 %)	38 (70.4 %)	31 (57.4 %)	0.36
DM (n, %)	16 (30.2 %)	15 (28.3 %)	16 (29.6 %)	7 (13.0 %)*	0.05
Fasting PG (mg/dL)	111.2 ± 10.4	109.9 ± 10.8	105.8 ± 11.1^†^	104.5 ± 9.1	0.009
Absolute Δ	−1.0 (−6.0, 4.0)		−1.0 (−5.0, 4.0)		0.69
PG-1h (mg/dL)	200.3 ± 32.9	197.0 ± 33.3	190.9 ± 34.4	172.9 ± 26.8**	0.0001
Absolute Δ	−5.0 (−23.0, 14.0)		−15.5 (−33.0, 1.0)		0.006
PG-2h (mg/dL)	185.1 ± 25.5	180.1 ± 30.8	179.7 ± 32.5	162.2 ± 32.9**	0.004
Absolute Δ	−9.0 (−21.3, 19.3)		−18.0 (−33.0, 0.0)		0.03
AUC-PG	348.5 ± 42.1	342.0 ± 46.9	333.2 ± 48.9	306.4 ± 41.4**	0.0001
Absolute Δ	−7.0 (−13.3, 2.1)		−22.3 (−45.0, 10.0)		0.002
Incremental glucose peak (mg/dL)	95.0 ± 27.7	92.1 ± 29.1	90.9 ± 30.3	75.4 ± 26.2**	0.002
Absolute Δ	−6.0 (−19.3, 15.3)		−16.5 (−33.0, −2.0)		0.006
Fasting IRI (µU/mL)	6.4 ± 3.3	6.1 ± 2.9	6.6 ± 3.4	6.5 ± 3.0	0.49
Absolute Δ	−0.1 (−1.4, 0.7)		−0.2 (−2.0, 1.8)		0.63
IRI-1h (µU/mL)	53.9 ± 29.7	50.7 ± 25.2	54.3 ± 32.2	57.3 ± 29.8	0.28
Absolute Δ	0.1 (−10.6, 10.4)		0.7 (−7.0, 13.1)		0.27
IRI-2h (µU/mL)	64.4 ± 30.2	58.6 ± 27.2	61.8 ± 35.0	60.1 ± 30.5	0.78
Absolute Δ	−6.0 (−15.3, 5.2)		2.5 (−15.5, 10.9)		0.2
AUC-IRI	89.3 ± 42.1	83.0 ± 36.6	88.5 ± 48.3	89.7 ± 42.4	0.38
Absolute Δ	−3.7 (−19.5, 8.6)		4.9 (−7.4, 11.4)		0.08
AUC-IRI/AUC-PG	0.23 (0.18, 0.35)	0.24 (0.17, 0.29)	0.24 (0.17, 0.33)	0.27 (0.19, 0.36)**	0.06
Absolute Δ	−0.01 (−0.04, 0.03)		0.02 (−0.01, 0.06)		0.003
Fasting TG (mg/dL)	123.3 ± 59.2	120.8 ± 62.2	137.8 ± 67.6	103.0 ± 38.1**	0.10
Absolute Δ	−3.0 (−35.0, 19.0)		−24.0 (−54.0, −3.0)		0.005
TG-1h (mg/dL)	138.5 ± 61.8	136.2 ± 64.0	150.7 ± 64.7	121.1 ± 37.7**	0.17
Absolute Δ	0.0 (−29.5, 16.5)		−20.5 (−57.0, 4.0)		0.01
TG-2h (mg/dL)	168.9 ± 74.8	171.6 ± 89.3	198.3 ± 78.7	150.8 ± 44.7**	0.14
Absolute Δ	−2.0 (−33.8, 27.3)		−42.0 (−80.0, 10.0)		0.0001
AUC-TG	285.0 ± 126.5	282.0 ± 137.3	316.8 ± 135.1	250.0 ± 76.3**	0.13
Absolute Δ	−5.5 (−60.5, 37.3)		−51.5 (−127.0, 5.0)		0.002
Incremental TG peak (mg/dL)	45.2 ± 31.2	51.9 ± 43.7	60.5 ± 29.8†	47.8 ± 24.7*	0.54
Absolute Δ	4.0 (−12.0, 21.3)		−11.5 (−24.0, 3.0)		0.005

P values represent comparison of each values between groups at 6 months except for absolute Δ

As for absolute Δ of each values, P values represent comparison between the two groups

Values are presented as mean ± standard deviation or medians and interquartile ranges, as indicated

*DM* diabetes mellitus, *IGT* impaired glucose tolerance, *NGT* normal glucose toleransce, *PG* plasma glucose, *AUC* area under the response curve, *IRI* immune reactive insulin, *TG* triglyceride

^†^ P < 0.05 vs baseline values of non-EPA group

* P < 0.01 vs baseline, ** P < 0.0001 vs baseline

Significant reductions in AUC-PG and incremental glucose peak were only observed for the EPA group (Table 4; Fig. 3). Despite significant improvements in AUC-PG, the EPA group exhibited a mild increase in AUC-IRI, while the non-EPA group exhibited decreased AUC-IRI accompanied by AUC-PG decrease (Fig. 3). Thus, the AUC-IRI/AUC-PG ratio, which indicated postprandial insulin secretion ability, significantly increased only in the EPA group.

Compared to baseline data, the EPA group exhibited significant reductions in TG-1h, TG-2h, and fasting TG levels. Furthermore, incremental TG peak and AUC-TG decreased only in the EPA group.

As for the comparison of absolute change from baseline between the EPA and non-EPA groups, the changes of PG-1h, PG-2h, AUC-PG, incremental glucose peak, fasting TG, TG-1h, TG-2h, AUC-TG, and incremental TG peak were significantly higher in the EPA group than the non-EPA group. Furthermore, the changes of AUC-IRI/AUC-PG ratio from baseline was significantly larger in the EPA group than the non-EPA group.

The multiple regression analysis showed that EPA treatment and lower baseline PG levels were independent factor for predicting improvement of incremental glucose peak (Additional file 1: Table S1). In order to adjust the difference of baseline PG levels between the two groups, additional analysis was performed among patients with similar baseline PG levels of ≤110 mg/dL (Additional file 2: Table S2). The absolute changes of incremental glucose and TG peak in the EPA group remained larger than those in the non-EPA group, and also the change of AUC-IRI/AUC-PG ratio in the EPA group was significantly higher than that in the non-EPA group.

### Comparing inflammatory markers and endothelial function

As shown in Table 3, the improvement of C-reactive protein levels was significantly higher in the EPA group than in the non-EPA group (P = 0.05). Although both groups exhibited impaired endothelial function at baseline, the EPA group exhibited a significant improvement in %FMD after 6 months of treatment. In contrast, endothelial dysfunction was unchanged in the non-EPA group. The results of the univariate and multivariate regression analyses for predicting improvements in %FMD are shown in Table 5. In the EPA group, improvements in the TG/HDL-C ratio and incremental TG peak were independent predictors of %FMD improvements.

**Table 5:** Regression analysis for predicting % flow-mediated dilatation improvement

Variable	Non-EPA group (n = 53)				EPA group (n = 54)			
	Univariate		Multivariate		Univariate		Multivariate	
	t	P value	t	P value	t	P value	t	P value
Age	2.865	0.006	2.505	0.01	0.085	0.93	0.013	0.99
Sex	−1.640	0.11	−1.038	0.30	−0.106	0.92	0.055	0.96
Changes in weight	−0.123	0.90			−0.045	0.95		
Changes in fasting PG	0.837	0.41			1.531	0.13		
Changes in fasting IRI	0.584	0.56			1.093	0.28		
Changes in Hemoglobin A1c	0.437	0.66			0.399	0.69		
Changes in 1,5-anhydro-glucitol	−1.463	0.15			1.542	0.13		
Changes in HOMA-R	0.027	0.98			0.596	0.55		
Changing in PG-1h	−0.675	0.50			−1.305	0.20		
Changing in PG-2h	1.776	0.08	−1.092	0.28	−0.923	0.36		
Changing in AUC-PG	0.049	0.96			−0.945	0.35		
Changing in incremental glucose peak	−0.030	0.98			−2.227	0.03	−1.054	0.30
Changing in IRI-1h	−0.896	0.37			0.008	0.99		
Changing in IRI-2h	1.549	0.13			−1.156	0.25		
Changing in AUC-IRI	−0.154	0.88			−0.511	0.61		
Changing in AUC-IRI/AUC-PG	−0.276	0.78			−0.001	0.99		
Changes in total cholesterol	−0.680	0.50			−0.349	0.73		
Changes in LDL cholesterol	−0.531	0.60			0.280	0.78		
Changes in HDL cholesterol	−0.085	0.93			0.328	0.74		
Changes in TG	−1.925	0.05	0.274	0.79	−2.062	0.04		
Changes in log TG/HDL ratio	−1.234	0.22			−2.009	0.05	−2.001	0.05
Changing in TG2h	1.234	0.22			−3.842	0.0003		
Changing in AUC-TG	−2.140	0.03	−0.288	0.77	−2.727	0.009		
Changing in incremental TG peak	−1.413	0.16			−4.337	<0.0001	−3.560	0.0008
Changes in remnant-like particle cholesterol	−2.282	0.03	−2.022	0.05	−0.369	0.71		
Changes in EPA	−0.269	0.79			−0.213	0.83		
Changes in AA	−0.174	0.86			−0.142	0.89		
Changes in docosahexaenoic acid	−0.752	0.46			−1.290	0.20		
Changes in EPA/AA	−0.469	0.64			0.109	0.91		
Changes in C-reactive protein	−0.560	0.58			0.328	0.74		

*PG* plasma glucose, *IRI* immune reactive insulin, *HOMA*-*R* homeostasis model assessment ratio, *AUC* area under the response curve, *LDL* low-density lipoprotein, *HDL* high-density lipoprotein, *TG* triglyceride, *EPA* eicosapentaenoic acid, *AA* arachidonic acid

## Discussion

Six months of EPA treatment improved not only fasting atherogenic lipid derangements and postprandial hypertriglyceridemia, but also postprandial hyperglycemia and insulin secretion ability. The simultaneous improvements in several metabolic abnormalities were associated with improvements in concomitant endothelial dysfunction among newly diagnosed IGM patients with CAD who received EPA.

### Effects of EPA on postprandial hyperglycemia

Accumulating data from animal models confirm that n-3 PUFA have numerous beneficial effects on health and diseases, such as anti-arrhythmic, vasodilatory, anti-inflammatory, anti-thrombotic, and lipid lowering actions [21–23]. N-3 PUFA also have a number of metabolic effects including improvement in insulin secretion, sensitivity and anti-obesity action [7, 8]. The recent experiments have revealed that n-3 PUFA act as ligands of several G-protein- coupled receptors (GPCRs) including GPCR 40 on pancreatic β cells and GPCR 120 on enteroendocrine cells of gastrointestinal tract [24, 25]. These actions enforce the beneficial effects on insulin secretion and sensitivity, and glucose homeostasis. However, many previous clinical trials have reported inconsistent results regarding the association of n-3 PUFA intake with the incidence of DM and glycemic control [8–10]. In the present study, we found that EPA treatment resulted in significant improvements in postprandial insulin secretion and glucose homeostasis among patients with IGM and significantly ameliorated the development of DM. Although we did not assess glucagon-like peptide-1 concentrations, we speculate that EPA-activated GPCRs might be actively involved in postprandial insulin secretion. These results suggest that EPA might provide a greater protective effect against developing DM among pre-diabetic patients. The results from the Japan EPA Lipid Intervention Study (JELIS) trial [11] support this possibility, as long-term treatment with EPA resulted in a significant 33 % reduction in the incidence of new-onset DM among patients with IGM (U.S. Patent Application Publication No. US2015/0250754 A1).

Although a few previous studies have reported that n-3 PUFA improved insulin resistance [10], we did not observe any significant improvement in HOMA-R for the EPA group. There are two possible explanations for this finding. First, our mean pre-treatment HOMA-R was 1.6, which was near the upper limit for normal HOMA-R results in the Japanese population, and might preclude the detection of any significant change. Second, EPA treatment did not alter fasting glycemic control at 6 months. Because HOMA-R was calculated using fasting PG and insulin levels, EPA treatment did not significantly improve HOMA-R. Many human studies have also failed to demonstrate that n-3 PUFA exert a protective effect on insulin sensitivity [8, 9]. Thus, future studies should be warranted in selected study populations.

Most trials have involved the use of diets supplemented by intake of fish, fish oil or capsule containing fish oil extracts. These contains a number of other fatty acids and different components. Thus, an evaluation of the specific effects of each n-3 PUFA was not possible. We adopted highly purified EPA ethyl ester, which was approved by Japan’s Ministry of Health, Labour and Welfare for the treatment of peripheral artery disease and hyperlipidemia, to examine its effects on postprandial glucose and insulin metabolism [11, 12].

### Effects of EPA on atherogenic dyslipidemia and postprandial hypertriglyceridemia

Despite best evidence-based statin therapy, it is clear that there persists unacceptably high residual cardiovascular events [26]. The importance of atherogenic dyslipidemia, defined as elevated TG-rich lipoproteins and their remnants and low HDL-C, has been recognized as a key driver of residual cardiovascular risk in individuals with metabolic disease, even if LDL cholesterol are well controlled [26, 27]. Type 2 DM commonly represents elevated TG, low HDL-C, and the predominance of small dense LDL particles due to insulin resistance [28]. Thereby, patients with DM are thought to belong to the highest cardiovascular disease risk category despite being treated by statin [1, 2, 29].

Several methods have been developed for the measurement of atherogenic small dense LDL levels, including ultracentrifugation, gradient gel electrophoresis, nuclear magnetic resonance spectroscopy and heparin magnesium precipitation methods [30, 31]. However, there is no standard procedure for clinical practice. Maruyama et al. demonstrated that the TG/HDL-C ratio was associated with LDL particle size [17]. Previous reports revealed that a high TG/HDL-C ratio was associated with cardiovascular disease and all-cause death among patients with DM or metabolic syndrome [17, 32, 33] and the amelioration of this abnormality was associated with improvements in endothelial dysfunction [34]. Thus, high TG/HDL-C ratio was regarded as an important residual cardiovascular risk and a therapeutic target for decreasing cardiovascular events in DM patients. Therefore, we used the TG/HDL-C ratio as the marker of small dense LDL particle in this study.

Long-term ingestion of fish oils (which include n-3 PUFA) by normal volunteers and patients with hypertriglyceridemia dramatically reduced fasting TG levels and postprandial hyperlipidemia that were produced by fat loading [35, 36]. In this context, n-3 PUFA have been suggested to predominantly suppress both hepatic and intestinal ApoB secretion and synthesis. EPA treatment also reduced TG levels with minimal changes in HDL-C levels for both healthy volunteers and patients with CAD [11, 21–23] and significantly decreased postprandial TG elevation and concomitantly improved postprandial endothelial dysfunction among healthy individuals [35]. The present study also revealed that 6 months of EPA treatment significantly reduced postprandial hypertriglyceridemia (incremental TG peak and AUC-TG) and remnant-like particle cholesterol as well as fasting TG levels and TG/HDL-C ratio, and that these improvements were associated with improvements in endothelial dysfunction among patients with newly diagnosed IGM. Therefore, these results suggest that EPA has beneficial effects on postprandial hypertriglyceridemia and other atherogenic dyslipidemia, such as remnant-like particle cholesterol and small dense LDL particles, and may significantly contribute to the residual risk reduction of cardiovascular events among patients with IGM. Indeed, JELIS trials have demonstrated that EPA treatment (1800 mg/day) significantly reduced the risk of major coronary events during an average follow-up of 4.6 years, and sub-analyses revealed that EPA treatment was especially beneficial among patients with IGM or patients with high TG and low HDL-C levels [11, 37, 38].

### Effects of EPA on FMD

Endothelial dysfunction contributes to the onset and progression of atherosclerosis [39]. Furthermore, endothelial dysfunction is associated with many cardiovascular risk factors, such as dyslipidemia, hypertension, DM, and obesity. Moreover, postprandial hyperglycemia and hypertriglyceridemia cause temporal and persistent endothelial dysfunction, and are thought to be associated with increased risk of CAD, especially among pre-diabetic and diabetic patients [18, 35]. The pathophysiology of FMD, based on reactive hyperemia, has extensively been studied and a previous large-scale population-based study demonstrated that brachial FMD predicted the incidence of cardiovascular events among adults [20]. Therefore, we selected percent change in FMD as the surrogate marker for the risk of future cardiovascular events.

EPA or re-composition of plasma membrane fatty acids following EPA administration (high EPA/AA ratio) directly improves endothelial dysfunction and provides a vasodilatory effect among patients with CAD and hyperlipidemia [40, 41]. Moreover, EPA upregulates endothelial nitric oxide synthase and augments nitric oxide production through the activation and translocation of endothelial nitric oxide synthase in endothelial cells [42].

In the present study, we measured plasma EPA levels and the EPA/AA ratio to monitor the bioavailability of EPA. There is accumulating evidence that blood EPA levels and the EPA/AA ratio predict the risk of cardiovascular events among the general population and patients with CAD [43]. It appears that EPA possesses a variety of biological actions including anti-inflammatory and anti-thrombotic effects, which may contribute to the prevention of cardiovascular events [22, 23, 44, 45]. Indeed, the present study also revealed improvements in inflammatory marker by using EPA and then improved endothelial dysfunction. Furthermore, based on EPA’s beneficial effects on metabolic derangements and endothelial protection, it appears that n-3 PUFA may reduce the risk of developing DM and/or cardiovascular events among patients with IGM.

### Effects on EPA on fasting glucose control

Most observational studies have reported an association between glucose control and cardiovascular disease [46]. Furthermore, several randomized controlled studies of patients with type 2 DM have demonstrated that intensive glucose control was associated with a significant reduction in the rate of major cardiovascular events [47]. Therefore, glycemic control plays an important role in preventing CAD among patients with diabetes. However, the present study found no changes in the EPA group’s fasting PG, IRI, ad hemoglobin A1c levels after 6 months of treatment. In this context, the STOP-NIDDM trial reported that acarbose (an α-glucosidase inhibitor) reduced the risk of cardiovascular events among patients with IGT, although these patients did not experience any changes in their fasting PG [48]. The authors speculated that a reduction in postprandial hyperglycemia and TG levels might reduce oxidative stress and endothelial dysfunction, which would subsequently be associated with the prevention of cardiovascular events.

### Study limitations

Several limitations in the present study warrant consideration. First, this study was open-label, single-blinded and number of participants was relatively small. Second, baseline PG level of the EPA group was significantly lower than that of the non-EPA group and multiple regression analysis for predicting incremental glucose peak improvement revealed lower baseline PG levels and EPA treatment. Whether the difference of baseline PG level between the two groups might influence the results of the present study was tested by additional statistical analysis. The improvement of incremental glucose and TG peak in the EPA group remained larger than that in the non-EPA group and the change of AUC-IRI/AUC-PG ratio in the EPA group was significantly higher than that in non-EPA group even among patients with similar baseline PG levels ≤110 mg/dL. Therefore, these results supported the improvements of postprandial hyperglycemia, hypertriglyceridemia and insulin secretory ability might be due to an effect of EPA. Third, cohort studies have reported that n-3 PUFA consumption was inversely associated with the incidence of DM in Asia, whereas it was positively associated in North America and Europe [4]. Thus, our results may not generalize to other geographical or racial populations. Fourth, docosahexaenoic acid has an in vitro agonistic effect on GPCR 40 that is stronger than the effect of EPA [24], and both EPA and docosahexaenoic acid have numerous distinct biological effects. Therefore, future studies are needed to explore the effects of docosahexaenoic acid on the incidences of DM and glucose homeostasis.

## Conclusions

In conclusion, EPA corrected not only postprandial hypertriglyceridemia but also postprandial hyperglycemia and insulin secretion ability. This amelioration of metabolic abnormalities was associated with improvements in concomitant endothelial dysfunction among newly diagnosed IGM patients with CAD. Though lifestyle changes remain a cornerstone in all strategy for prevention of DM, this study suggests that addition of EPA could accelerate the prevention of DM and CAD.

## Additional files

10.1186/s12933-016-0437-y Multiple regression analysis for predicting incremental glucose peak improvement.
10.1186/s12933-016-0437-y Comparison of cookie meal test data between baseline and 6 months, and comparison of absolute change from baseline among patients with baseline plasma glucose <110 mg/dL.

## Abbreviations

DM: diabetes mellitus
CAD: coronary artery disease
PUFA: polyunsaturated fatty acids
EPA: eicosapentaenoic acids
IGM: impaired glucose metabolism
PG: plasma glucose
TG: triglyceride
OGTT: oral glucose tolerance test
IGT: impaired glucose tolerance
IRI: immune reactive insulin
HDL-C: high-density lipoprotein cholesterol
LDL: low-density lipoprotein
CMT: cookie meal test
AUC: area under the response curve
HOMA-R: homeostasis model assessment ratio
FMD: flow-mediated dilatation
AA: arachidonic acid
GPCR: G-protein coupled receptor
