# Supplementary material for: Effects of 6-month eicosapentaenoic acid treatment on postprandial hyperglycemia, hyperlipidemia, insulin secretion ability, and concomitant endothelial dysfunction among newly-diagnosed impaired glucose metabolism patients with coronary artery disease. An open label, single blinded, prospective randomized controlled trial
Source: Cardiovasc Diabetol. 2016 Aug 26;15(1):121. doi: 10.1186/s12933-016-0437-y (PMC5002116; doi:10.1186/s12933-016-0437-y)
Supplement: Supplementary file 1 — 10.1186/s12933-016-0437-y Multiple regression analysis for predicting incremental glucose peak improvement. [file 12933_2016_437_MOESM1_ESM.docx]

| **Table S1, multiple regression analysis for predicting incremental glucose peak improvement** | | |
| --- | --- | --- |
| Variable | multivariate | |
|  | t | p-value |
| age | -0.121 | 0.90 |
| sex | -0.484 | 0.63 |
| Baseline body mass index | -0.563 | 0.57 |
| Use of EPA ethyl ester | -2.287 | 0.02 |
| Baseline Fasting PG | 2.339 | 0.01 |
| Baseline EPA/AA ratio | 1.190 | 0.24 |

EPA; eicosapentaenoic acid, PG; plasma glucose, AA; arachidonic acid,
