# Supplementary material for: Effects of 6-month eicosapentaenoic acid treatment on postprandial hyperglycemia, hyperlipidemia, insulin secretion ability, and concomitant endothelial dysfunction among newly-diagnosed impaired glucose metabolism patients with coronary artery disease. An open label, single blinded, prospective randomized controlled trial
Source: Cardiovasc Diabetol. 2016 Aug 26;15(1):121. doi: 10.1186/s12933-016-0437-y (PMC5002116; doi:10.1186/s12933-016-0437-y)
Supplement: Supplementary file 2 — 10.1186/s12933-016-0437-y Comparison of cookie meal test data between baseline and 6 months, and comparison of absolute change from baseline among patients with baseline plasma glucose <110 mg/dL. [file 12933_2016_437_MOESM2_ESM.docx]

| **Table S2. Comparison of cookie meal test data between baseline and 6 months, and comparison of absolute change from baseline among patients with baseline plasma glucose <110 mg/dL.** | | | | | |
| --- | --- | --- | --- | --- | --- |
| Variable | non-EPA group　(n = 25) | | EPA group　(n = 37) | | P-value |
|  | Baseline | 6-month | Baseline | 6-month |  |
| Glucose tolerance test |  |  |  |  |  |
| NGT (n, %) | 0 (0.0%) | 1 (4.0%) | 0 (0.0%) | 15 (40.5%)** | 0.001 |
| IGT (n, %) | 18 (72.0%) | 21 (84.0%) | 27 (73.0%) | 20 (54.1%) | 0.02 |
| DM (n, %) | 7 (28.0%) | 3 (12.0%) | 10 (27.0%) | 2 (5.4%)* | 0.35 |
| Fasting PG (mg/dL) | 101.5±5.6 | 101.4±7.4 | 99.2±5.1 | 101.1±7.5 | 0.28 |
| Absolute Δ | 0.0 (-4.3, 5.0) | | 1.0 (-1.0, 5.3) | | 0.55 |
| PG-1h (mg/dL) | 188.7±27.7 | 181.8±25.3 | 181.4±32.2 | 164.6±22.3** | 0.007 |
| Absolute Δ | -5.0 (-23.0, 14.0) | | -15.5 (-33.0, 1.0) | | 0.006 |
| PG-2 h (mg/dL) | 181.0±26.8 | 170.3±29.0* | 172.6±25.9 | 149.7±24.5** | 0.005 |
| Absolute Δ | -16.0 (-21.0, 5.0) | | -22.0 (-32.5, -9.0) | | 0.08 |
| AUC-PG | 325.6±33.0 | 314.9±33.8* | 316.9±42.6 | 289.5±32.5** | 0.006 |
| Absolute Δ | -11.3 (-24.0, 2.5) | | -23.0 (-46.8, -10.8) | | 0.02 |
| Incremental glucose peak (mg/dL) | 91.8±22.6 | 82.8±26.2* | 86.2±29.1 | 66.2±21.1** | 0.01 |
| Absolute Δ | -10.5 (-24.0, 2.0) | | -20.5 (-34.0, -7.0) | | 0.04 |
| Fasting IRI (µU/mL) | 6.2±3.1 | 6.4±2.5 | 5.8±3.2 | 6.3±3.2 | 0.88 |
| Absolute Δ | -0.1 (-0.5, 0.8) | | 0.8 (-1.0, 2.4) | | 0.43 |
| IRI-1h (µU/mL) | 57.9±29.4 | 50.2±21.5 | 53.1±32.6 | 57.0±28.0 | 0.34 |
| Absolute Δ | -7.6 (-14.4, 10.1) | | 4.0 (-6.0, 15.2) | | 0.05 |
| IRI-2h (µU/mL) | 69.4±33.2 | 60.1±29.7* | 62.6±40.0 | 54.1±27.1* | 0.43 |
| Absolute Δ | -10.2 (-24.6, 0.3) | | -4.3 (-23.3, 9.1) | | 0.60 |
| AUC-IRI | 95.6±41.9 | 83.4±33.4* | 87.3±52.0 | 87.1±39.1 | 0.71 |
| Absolute Δ | -8.3 (-21.0, 5.4) | | 5.5 (-8.8, 14.5) | | 0.04 |
| AUC-IRI/ AUC-PG | 0.26 (0.18, 0.36) | 0.26 (0.20, 0.31) | 0.24 (0.17, 0.33) | 0.26 (0.22, 0.37)** | 0.39 |
| Absolute Δ | -0.03 (-0.06, 0.02) | | 0.03 (-0.01, 0.08) | | 0.006 |
| Fasting TG (mg/dL) | 126.2±52.0 | 122.6±63.5 | 133.3±69.0 | 103.2±41.0** | 0.16 |
| Absolute Δ | -3.0 (-35.0, 22.0) | | -22.0 (-42.5, -0.5) | | 0.09 |
| TG-1h (mg/dL) | 141.9±60.9 | 134.9±61.2 | 145.5±65.4 | 119.9±40.8** | 0.03 |
| Absolute Δ | -1.5 (-29.0, 10.0) | | -19.5 (-46.5, 4.5) | | 0.09 |
| TG-2h (mg/dL) | 173.4±73.0 | 171.6±89.3 | 193.5±76.5 | 147.0±48.4** | 0.19 |
| Absolute Δ | 0.0 (-33.0, 21.0) | | -39.0 (-62.5, -17.0) | | 0.005 |
| AUC-TG | 289.4±119.3 | 276.5±127.2 | 308.9±135.0 | 245.0±82.9** | 0.25 |
| Absolute Δ | -5.5 (-60.5, 37.3) | | -51.5 (-127.0, 5.0) | | 0.04 |
| Incremental TG peak (mg/dL) | 47.6±29.1 | 46.0±37.8 | 60.3±29.7 | 43.8±25.8** | 0.79 |
| Absolute Δ | -4.0 (-12.0, 7.3) | | -18.0 (-28.0, -4.0) | | 0.04 |

P-values represent comparison of each values between groups at 6 months except for absolute Δ.

As for absolute Δ of each values, P-values represent comparison between the two groups.

Values are presented as means ± standard deviation or medians and interquartile ranges, as indicated.

†P<0.05 vs baseline values of non-EPA group

* P<0.01 vs baseline, ** P<0.0001 vs baseline.

DM; diabetes mellitus, IGT; impaired glucose tolerance, NGT; normal glucose toleransce

PG; plasma glucose, AUC; area under the response curve, IRI; immune reactive insulin, TG; triglyceride,
